# Matrix metalloproteinases in human melanoma cell lines and xenografts: increased expression of activated matrix metalloproteinase-2 (MMP-2) correlates with melanoma progression

**DOI:** 10.1038/sj.bjc.6690763

**Published:** 1999-11

**Authors:** U B Hofmann, J R Westphal, E T Waas, A J W Zendman, I M H A Cornelissen, D J Ruiter, G N P van Muijen

**Affiliations:** Departments of Pathology andSurgery, University Hospital, Nijmegen, The Netherlands; Department of Dermatology, University Hospital, Würzburg, Germany

**Keywords:** matrix metalloproteinase (MMP), tissue inhibitor of matrix metalloproteinases (TIMP), melanoma, xenograft, invasion, metastasis

## Abstract

Matrix metalloproteinases (MMPs) and their tissue inhibitors (TIMPs) are involved in tumour progression and metastasis. In this study, we investigated the in vitro and in vivo expression patterns of MMP-1, MMP-2, MMP-3, MMP-9, TIMP-1 and TIMP-2 mRNA and protein in a previously described human melanoma xenograft model. This model consists of eight human melanoma cell lines with different metastatic behaviour after subcutaneous (s.c.) injection into nude mice. MMP-1 mRNA was detectable in all cell lines by reverse transcription polymerase chain reaction (RT-PCR), but the expression was too low to be detected by Northern blot analysis. No MMP-1 protein could be found using Western blotting. MMP-2 mRNA and protein were present in all cell lines, with the highest expression of both latent and active MMP-2 in the highest metastatic cell lines MV3 and BLM. MMP-3 mRNA was expressed in MV3 and BLM, and in the non-metastatic cell line 530, whereas MMP-3 protein was detectable only in MV3 and BLM. None of the melanoma cell lines expressed MMP-9. TIMP-1 and TIMP-2 mRNA and protein, finally, were present in all cell lines. A correlation between TIMP expression level and metastatic capacity of cell lines, however, was lacking. MMP and TIMP mRNA and protein expression levels were also studied in s.c. xenograft lesions derived from a selection of these cell lines. RT-PCR analysis revealed that MMP-1 mRNA was present in MV3 and BLM xenografts, and to a lesser extent in 530. Positive staining for MMP-1 protein was found in xenograft lesions derived from both low and high metastatic cell lines, indicating an in vivo up-regulation of MMP-1. MMP-2 mRNA was detectable only in xenografts derived from the highly metastatic cell lines 1F6m, MV3 and BLM. In agreement with the in vitro results, the highest levels of both latent and activated MMP-2 protein were observed in MV3 and BLM xenografts. With the exception of MMP-9 mRNA expression in 530 xenografts, MMP-3, MMP-9, and TIMP-1 mRNA and protein were not detectable in any xenograft, indicating a down-regulated expression of MMP-3 and TIMP-1 in vivo. TIMP-2 mRNA and protein were present in all xenografts; interestingly, the strongest immunoreactivity of tumour cells was found at the border of necrotic areas. Our study demonstrates that of all tested components of the matrix metalloproteinase system, only expression of activated MMP-2 correlates with increased malignancy in our melanoma xenograft model, corroborating an important role of MMP-2 in human melanoma invasion and metastasis. © 1999 Cancer Research Campaign


*British Journal of Cancer* (1999) **81**(5), 774–782
© 1999 Cancer Research Campaign
Article no. bjoc.1999.0763

### Matrix metalloproteinases in human melanoma cell lines and xenografts: increased expression of activated matrix metalloproteinase-2 (MMP-2) correlates with melanoma progression

**UB Hofmann[1,3], JR Westphal[1], ET Waas[2], AJW Zendman[1], IMHA Cornelissen[1], DJ Ruiter[1] and GNP van Muijen[1]**

Departments of [1]Pathology and [2]Surgery, University Hospital, Nijmegen, The Netherlands; [3]Department of Dermatology, University Hospital, Würzburg, Germany

**Summary** Matrix metalloproteinases (MMPs) and their tissue inhibitors (TIMPs) are involved in tumour progression and metastasis. In this study, we investigated the in vitro and in vivo expression patterns of MMP-1, MMP-2, MMP-3, MMP-9, TIMP-1 and TIMP-2 mRNA and protein in a previously described human melanoma xenograft model. This model consists of eight human melanoma cell lines with different metastatic behaviour after subcutaneous (s.c.) injection into nude mice. MMP-1 mRNA was detectable in all cell lines by reverse transcription polymerase chain reaction (RT-PCR), but the expression was too low to be detected by Northern blot analysis. No MMP-1 protein could be found using Western blotting. MMP-2 mRNA and protein were present in all cell lines, with the highest expression of both latent and active MMP-2 in the highest metastatic cell lines MV3 and BLM. MMP-3 mRNA was expressed in MV3 and BLM, and in the non-metastatic cell line 530, whereas MMP-3 protein was detectable only in MV3 and BLM. None of the melanoma cell lines expressed MMP-9. TIMP-1 and TIMP-2 mRNA and protein, finally, were present in all cell lines. A correlation between TIMP expression level and metastatic capacity of cell lines, however, was lacking. MMP and TIMP mRNA and protein expression levels were also studied in s.c. xenograft lesions derived from a selection of these cell lines. RT-PCR analysis revealed that MMP-1 mRNA was present in MV3 and BLM xenografts, and to a lesser extent in 530. Positive staining for MMP-1 protein was found in xenograft lesions derived from both low and high metastatic cell lines, indicating an in vivo up-regulation of MMP-1. MMP-2 mRNA was detectable only in xenografts derived from the highly metastatic cell lines 1F6m, MV3 and BLM. In agreement with the in vitro results, the highest levels of both latent and activated MMP-2 protein were observed in MV3 and BLM xenografts. With the exception of MMP-9 mRNA expression in 530 xenografts, MMP-3, MMP-9, and TIMP-1 mRNA and protein were not detectable in any xenograft, indicating a down-regulated expression of MMP-3 and TIMP-1 in vivo. TIMP-2 mRNA and protein were present in all xenografts; interestingly, the strongest immunoreactivity of tumour cells was found at the border of necrotic areas. Our study demonstrates that of all tested components of the matrix metalloproteinase system, only expression of activated MMP-2 correlates with increased malignancy in our melanoma xenograft model, corroborating an important role of MMP-2 in human melanoma invasion and metastasis. © 1999 Cancer Research Campaign

**Keywords:** matrix metalloproteinase (MMP); tissue inhibitor of matrix metalloproteinases (TIMP); melanoma; xenograft; invasion; metastasis

Degradation and remodeling of the extracellular matrix (ECM) is an essential step in tumour cell migration, invasion and metastasis. This process is mediated mainly by two families of proteolytic enzymes, namely components of the plasminogen activation system, and the matrix metalloproteinases (MMPs) (Stetler Stevenson et al, 1993). MMPs are a rapidly growing family of structurally related enzymes that degrade components of the ECM. Based on their structure and substrate specificity, they are generally classified into different groups of closely related members, including collagenases (MMP-1, MMP-8, MMP-13), gelatinases (MMP-2 and MMP-9), stromelysins (MMP-3, MMP-10, MMP-11), metalloelastase (MMP-12), (MMP-18), (MMP-19), matrilysin (MMP-7), enamelysin (MMP-20), the newly discovered MMP-23, and five membrane-type MMPs. In contrast to the soluble MMPs, these recently discovered MT-MMPs possess a transmembrane domain, resulting in cell surface expression (Sato et al, 1994). Most MMPs are secreted from cells in latent forms (proMMPs). Conversion of proMMPs to functionally active forms requires a specific multistep activation process (Nagase, 1997). MMP activity is further modulated by a family of naturally occurring tissue inhibitors of matrix metalloproteinases (TIMPs), of which at least four different types (TIMP-1 to TIMP-4) have been identified (Gomez et al, 1997). TIMPs bind either proMMPs or active MMPs, thereby inhibiting the autocatalytic activation of latent enzymes, as well as the proteolytic capacity of active proteinases. The balance between levels of activated MMP and free inhibitors determines overall MMP activity (Gomez et al, 1997).

Expression of MMPs and TIMPs changes during the progression of normal cells towards malignancy. Elevated secretion of MMPs by tumour cells has been demonstrated in a variety of

*Received 10 February 1999*
*Revised 26 April 1999*
*Accepted 29 April 1999*

*Correspondence to:* UB Hofmann, Department of Pathology, University Hospital, P.O. Box 9101, 6500 HB Nijmegen, The Netherlands

**Table 1** Biological characteristics of human melanoma cell lines

| Cell lines | In vitro growth rate | In vivo growth rate[a] | Metastases formation[a] | Speed of metastases formation[a] |
|---|---|---|---|---|
| 530 | Slow | Slow | No | Not applicable |
| 1F6 | Slow | Medium | No | Not applicable |
| MV1 | Fast | Medium | Yes | Weeks/month |
| M14 | Fast | Medium | Yes | Weeks/month |
| Me57 | Fast | Medium | Yes | Weeks/month |
| 1F6m | Medium | Medium | Yes | Weeks/month |
| MV3 | Fast | Fast | Yes | Days/weeks |
| BLM | Fast | Fast | Yes | Days/weeks |

[a]After s.c. inoculation into nude mice.

**Table 2** Sequences of PCR primer sets used (Grant et al, 1996)

| Factor | Primer sequences | Product size |
|---|---|---|
| MMP-1 | 5′-CGACTCTAGAAACACAAGAGCAAGA-3′ (sense)<br>5′-AAGGTTAGCTTACTGTCACACGCTT-3′ (antisense) | 786-bp |
| MMP-2 | 5′-GTGCTGAAGGACACACTAAAGAAGA-3′ (sense)<br>5′-TTGCCATCCTTCTCAAAGTTGTAGG-3′ (antisense) | 580-bp |
| MMP-3 | 5′-AGATGCTGTTGATTCTGCTGTTGAG-3′ (sense)<br>5′-ACAGCATCAAAGGACAAAGCAGGAT-3′ (antisense) | 515-bp |
| MMP-9 | 5′-CACTGTCCACCCCTCAGAGC-3′ (sense)<br>5′-GCCACTTGTCGGCGATAAGG-3′ (antisense) | 243-bp |
| TIMP-1 | 5′-ATCCTGTTGTTGCTGTGGCTGATAG-3′ (sense)<br>5′-TGCTGGGTGGTAACTCTTTATTTCA-3′ (antisense) | 667-bp |
| TIMP-2 | 5′-AAACGACATTTATGGCAACCCTATC-3′ (sense)<br>5′-ACAGGAGCCGTCACTTCTCTTGATG-3′ (antisense) | 405-bp |
| $\beta_2$-m | 5′-GCTCTGTCTCTCGTGGTC-3′ (sense)<br>5′-GCATGGTCCACAGTTCTTG-3′ (antisense) | 136-bp |

cancers (Coussens and Werb, 1996; Chambers and Matrisian, 1997), and the resulting imbalance between MMPs and their specific inhibitors has been shown to play an important role in tumour growth and invasion (Stetler Stevenson et al, 1993; Chambers and Matrisian, 1997). In cultured human melanoma cells elevated expression of MMP-1, MMP-2, and MMP-9 has been shown to be correlated with migration and invasion (MacDougall et al, 1995; Ray and Stetler Stevenson, 1995; Durko et al, 1997). Increased expression of TIMP-1, TIMP-2 and TIMP-3 was able to inhibit these processes (Khokha et al, 1992; Montgomery et al, 1994; Imren et al, 1996; Ahonen et al, 1998; Valente et al, 1998). In human melanocytic lesions a positive correlation between tumour progression and MMP-2 expression level was demonstrated (Vaisanen et al, 1996, 1998). Increased expression of MMP-9, on the other hand, was found mainly in radial growth phase of primary melanoma, indicating that MMP-9 expression correlates with early invasion of melanoma (van den Oord et al, 1997).

In the present study we investigated the expression of MMP-1, -2, -3, -9, TIMP-1 and TIMP-2 mRNA and protein in a previously described xenograft model consisting of eight human melanoma cell lines with different metastatic capacity after subcutaneous (s.c.) injection into nude mice (van Muijen et al, 1991*a*, 1991*b*; Westphal et al, 1997). We show for the first time that increased expression of activated MMP-2 is correlated with melanoma progression, indicating that activated MMP-2 may be required for melanoma invasion and metastasis.

### MATERIALS AND METHODS

### Cell lines and culture conditions

Melanoma cell lines 530, 1F6, MV1, M14, Mel57, 1F6m, MV3 and BLM have been described previously (van Muijen et al, 1991*a*, 1991*b*). All cell lines were derived from surgically removed human metastases, and form local tumours after s.c. injection into nude mice. The xenografts differ in growth rate and capacity to form spontaneous lung metastases, as described previously (Westphal et al, 1997) (Table 1). Melanoma cell line M24met was kindly provided by Dr B Mueller (The Scripps Institute, La Jolla, CA, USA), and served as a positive control for MMP-1 expression. Human fibrosarcoma cell line HT1080 (American Type Culture Collection, Rockville, MD, USA) served as a positive control for MMP-9 expression. All cell lines were cultured in Dulbecco's modified Eagle's medium (DMEM) (Biowhittaker, MD, USA), supplemented with 10% fetal calf serum (FCS) (Integro, Zaandam, The Netherlands) and gentamycin (40 $\mu$g ml$^{-1}$, Gibco-BRL, Gaithersburg, MD, USA) in an atmosphere of 95% humidified air and 5% carbon dioxide at 37°C. Cells were detached from tissue-culture flasks with 0.05% trypsin, 0.02% EDTA and 0.1% glucose in phosphate-buffered saline (PBS) for 2 min at 37°C.

### Xenografts in nude mice and rats

Human melanoma cell lines were xenografted in BALB/c *nu/nu* mice kept under aseptic conditions according to NIH guidelines. A

© 1999 Cancer Research Campaign 

total of 3–5 × $10^6$ cells of human melanoma cell lines 530, 1F6, Mel57, 1F6m, MV3 and BLM were injected s.c. into the flank of nude mice. In order to be able to use mouse monoclonal antibodies for immunohistochemistry, 1F6 and BLM xenografts were also grown in nude rats. The animals were sacrificed when the tumour had a diameter of approximately 1 cm. Parts of the tumours were snap-frozen and stored in liquid nitrogen.

### RNA isolation

Total RNA from each cell line was isolated using the RNeasy Mini Kit (Qiagen, Hilden, Germany) according to the manufacturer's instructions. Typically, 1 × $10^7$ cells were used for each isolation, yielding 50–100 μg RNA. For RNA extraction from tissue samples, frozen sections from xenografts derived from melanoma cell lines were used. Total RNA was isolated after disrupting at least one 20-μm frozen section in 1 ml RNAzolB™ (Campro, Veenendaal, The Netherlands) using a pestle. Melanin and DNA were removed from RNA samples using the RNeasy kit.

### RT-PCR analysis

A total of 0.5 μg of RNA was reverse-transcribed with the 1st Strand cDNA Synthesis Kit for reverse transcription polymerase chain reaction (RT-PCR) (Boehringer Mannheim, Penzberg, Germany) using random hexadeoxynucleotide primers according to the manufacturer's instructions. One microlitre aliquots of the reverse-transcribed cDNA was subjected to PCR, using PCR buffer IV (20 mM $(NH_4)_2SO_4$, 75 mM Tris–HCl pH 9, 0.1% Tween) 0.2 M dNTPs, 2 pmoles of each primer, 1.5 mM magnesium chloride and 0.15 units of Thermoperfectplus DNA polymerase (Integro, Zaandam, The Netherlands). Water was added to a final volume of 25 μl. Each PCR was performed for 30 cycles (45 s at 94°C, 1 min at 59°C, 1 min 30 s at 72°C), preceeded by a denaturation step at 94°C for 3 min, and terminated with an elongation step at 72°C for 5 min. PCR products were visualized on 2% agarose gels containing ethidium bromide. As positive control for the RT-PCR procedure we used $\beta_2$-microglobulin ($\beta_2$-m). Incubations in which cDNA was omitted were used as negative controls. The PCR procedure was performed at least twice for each sample. The sequences of the human specific PCR primers are given in Table 2.

### Northern blot analysis

Aliquots of 5 μg of total RNA extracted from each cell line were electrophoresed on a 1% agarose gel containing MOPS/ paraformaldehyde and transferred to a nylon membrane (Boehringer Mannheim) by capillary blotting in 20 × standard saline citrate (SSC) according to standard protocols. Levels of 28S and 18S RNA were used as a control for RNA integrity and loading consistency. RNA was fixed to the membrane by baking for 30 min at 120°C. Probes for MMP-1 and MMP-2 were 1.4 kb and 2.6 kb cDNA fragments (gifts from Dr R Hanemaaijer (Gaubius Laboratory IVVO-TNO, Leiden, The Netherlands)), cloned in Bluescript KS vector and isolated from overnight cultures with the Wizard™ Miniprep Kit (Promega, Madison, WI, USA). DIG-labelled DNA probes were produced by performing PCR on cloned DNA fragments in the presence of 0.7 mM DIG-labelled dUTP in PCR DIG labelling mix (Boehringer Mannheim). DNA probes were purified with the QIAquick PCR Purification Kit (QIAGEN, Hilden, Germany) following the manufacturer's instructions. Blots were hybridized overnight at 50°C at a probe concentration of 40 ng $ml^{-1}$ in Easyhyb hybridization buffer (Boehringer Mannheim). Blots were washed twice for 5 min at room temperature in 2 × SSC 0.1% sodium dodecyl sulphate (SDS), once in 0.5 × SSC 0.1% SDS at 68°C for 15 min, and finally in 0.1 × SSC 0.1% SDS for 15 min at 68°C. The membranes were developed by chemiluminescence detection with CSPD or CDP-Star (Boehringer Mannheim), and exposed to Kodak X-Omat-AR film.

### Western blot analysis

Serum-free conditioned medium (SFCM) was generated by washing cells three times with PBS, followed by incubation overnight in serum-free medium. SFCM derived from 1 × $10^7$ cells from each cell line was used. Western blot analysis was performed according to standard protocols. Briefly, 12 μl samples of 10 times concentrated (Centricon 10 microconcentrator; Amicon, Beverly, MA, USA) SFCM were run on 10% polyacrylamide gel. Samples were incubated for 5 min at 95°C in the presence of 10% β-mercaptoethanol prior to electrophoresis. After electrophoresis, samples were electroblotted onto nitrocellulose (Schleier & Schuell, Dassel, Germany). Blots were blocked for 30 min in blocking solution (Boehringer Mannheim) at room temperature and then incubated overnight with 1 μg $ml^{-1}$ of primary antibody in blocking solution. After washing with PBS 0.05% Tween-20, membranes were incubated for 2 h at room temperature with a rabbit anti-mouse peroxidase-labelled secondary antibody (Dako, Denmark) in blocking solution. After additional washes, binding of peroxidase-labelled antibody was visualized by chemiluminescence (Boehringer Mannheim), according to the manufacturer's protocol.

### Gelatin zymography

Zymography was performed according to standard protocols. Aliquots of unconcentrated SFCM (5 μl) were diluted 1:1 in sample-buffer (62.5 mM Tris–HCl (pH 6.8), 8.8% glycerol, 2% (w/v) SDS, 0.05% bromophenol blue). After heating at 60°C for 20 min, samples were electrophoresed at 4°C on a 7.5% SDS-polyacrylamide gel containing 0.5 mg $ml^{-1}$ gelatin. After electrophoresis, gels were washed three times in 2.5% Triton X-100 for 10 min to remove SDS. After rinsing twice in substrate buffer (50 mM Tris–HCl, pH 7.8, containing 5 mM calcium chloride and 0.1% Triton X-100), the gels were incubated at 37°C for 18 h in the same buffer under gentle agitation. Gels were stained for 45 min in 40% methanol 10% glacial acetic acid containing 0.1% (w/v) Coomassie brilliant blue R250 and re-stained in the same solution without Coomassie brilliant blue. Activation of MMP-2 and MMP-9 was achieved by incubating samples before zymography with 1 mM p-aminophenylmercuric acetate (APMA) at 37°C for 2 h. Latent proMMP-2 ($M_r$ 66 000) and proMMP-9 ($M_r$ 92 000) as well as activated MMP-2 ($M_r$ 62 000) and MMP-9 ($M_r$ 84 000) were identified by comparing them to known gelatinolytic activity from conditioned medium from HT1080 cells (Sato et al, 1994) and control samples containing MMP-2 and MMP-9 protein (Oncogene Research Products, Cambridge, MA, USA) with and without APMA pretreatment. For zymography analysis of xenografts, 4 serial tissue cryostat sections (10-μm) were homogenized in 4 × sample-buffer, centifuged for 1 min at 10 000 ***g***, and

© 1999 Cancer Research Campaign pellets were incubated at 37°C for 30 min. Samples were mixed with distilled water (1:1) and heated at 60°C for 20 min. After centrifugation (1 min, 10 000 ***g***) pellets were discarded and supernatants were subjected to zymographic analysis. Zymography was performed at least twice for each sample.

### Antibodies

For Western blot analysis mouse monoclonal antibodies (mAbs) (Oncogene Research Products, Cambridge, MA, USA) against MMP-1 (clone 41-1E5), MMP-2 (clone 42-5D11), MMP-3 (clone 55-2A4), MMP-9 (clone 56-2A4), TIMP-1 (clone 7-6C1) and TIMP-2 (clone T2-101) were used. For immunohistochemistry a rabbit polyclonal antibody against human MMP-2 (# AB809, Chemicon International Inc, Temecula, CA, USA), sheep polyclonal antibodies against MMP-3 (# PH112, The Binding Site Limited, Birmingham, UK), MMP-9 (# PH163, The Binding Site Limited, Birmingham, UK), and mouse monoclonal antibodies (Oncogene Research Products, Cambridge, MA, USA) against MMP-1 (clone 41-1E5), TIMP-1 (clone 7-6C1) and TIMP-2 (clone T2-101) were used. A polyclonal rabbit antibody against human u-PA (Dako, Carpinteria, CA, USA) (Ferrier et al, 1998) was used as positive control.

### Immunohistochemistry

For immunohistochemistry 4-µm cryostat serial sections of xenograft lesions were used. At least three different xenografts per cell line were analysed. Sections were fixed for 10 min at room temperature in aceton with 0.15% hydrogen peroxide to inactivate endogenous peroxidase, followed by incubation overnight with primary antibodies in humidified chamber at 4°C. Subsequent steps were performed at room temperature. Prior to incubation with a biotinylated goat anti-rabbit, horse anti-mouse or goat anti-sheep secondary antibodies (Vector Laboratories, Burlingame, CA, USA) for 30 min, the avidin–biotin blocking kit (Vector Laboratories) was used according to the manufacturer's protocol. Forty-five minutes after addition of the avidin–biotin–peroxidase complex (Vectastain Elitekit, Vector Laboratories), sections were developed with amino-ethyl carbazol (AEC), and counterstained with Meyer's haematoxylin. An incubation where the first antibody was omitted served as negative control.

### RESULTS

We investigated the expression of MMP-1, MMP-2, MMP-3, MMP-9, TIMP-1 and TIMP-2 in a xenograft model consisting of eight human melanoma cell lines with different metastatic behaviour after s.c. inoculation into nude mice. Cell lines 530 and 1F6 are non- or sporadically metastasizing, whereas MV1, M14, Mel57, 1F6m, MV3 and BLM are frequently metastasizing. Of these six lines, MV3 and BLM tumours grow faster, and generate lung metastases much sooner compared to the other four cell lines (Westphal et al, 1997) (Table 1).

### MMP and TIMP mRNA in human melanoma cell lines

RT-PCR analysis detected MMP-1, MMP-2, TIMP-1 and TIMP-2 mRNA in all melanoma cell lines. MMP-3 mRNA was only produced by the very aggressive cell lines MV3 and BLM and, remarkably, also by the non-metastatic cell line 530. MMP-9 mRNA, on the other hand, was not detectable in any cell line; the 390-bp PCR band represents amplified DNA instead of RNA. A control RT reaction for MMP-9 without reverse transcriptase was performed (data not shown). The results of the RT-PCR analysis are shown in Figure 1. In order to make a quantitative comparison of MMP-1 and MMP-2 mRNA levels, we performed Northern blot analysis. MMP-1 mRNA levels were too low to be detected in Northern blotting, whereas MMP-2 mRNA levels were clearly elevated in the highly metastatic cell lines MV3 and BLM (Figure 2).

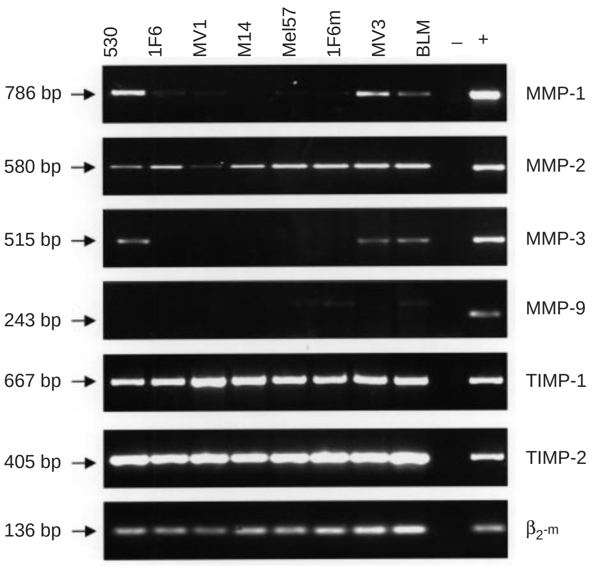

**Figure 1** RT-PCR analysis of MMP-1, MMP-2, MMP-3, MMP-9, TIMP-1 and TIMP-2 mRNA expression in human melanoma cell lines. An incubation without template cDNA was used as negative control. M24met or HT1080 mRNA were used as positive controls. PCR was performed at least twice for each component and each cell line. Lengths of the PCR products (bp) are indicated

### MMP and TIMP protein in human melanoma cell lines

MMP and TIMP protein levels were determined by Western blot analysis of 10 × concentrated SFCM derived from $1 \times 10^7$ cells (Figure 3). In agreement with our Northern blot results, MMP-1 protein was undetectable in our panel of cell lines. MMP-2 protein could be detected in all cell lines, but markedly increased in the highly metastatic cell lines MV3 and BLM. MMP-3 protein was present in SFCM of the cell lines MV3 and BLM but, despite MMP-3 mRNA expression, MMP-3 protein was not detectable in SFCM from cell line 530. In agreement with the results on mRNA no MMP-9 protein was detectable in any cell line. Western blots for TIMP-1 and TIMP-2 showed protein expression in all cell lines. Although the expression levels of TIMP-1 and TIMP-2 protein markedly differed among cell lines, there was no evident correlation with metastatic capacity in the xenograft model.

Activation status of MMP-2 was studied by zymography analysis of unconcentrated SFCM. Pretreatment of SFCM with p-APMA has been shown to induce the autocatalytic removal of the amino-terminal domain of the proMMPs, resulting in conversion to the active form. The latent proMMP-2 was detectable in all cell lines, but expression of functionally active MMP-2 was restricted to the highly metastatic cell lines MV3 and BLM.

© 1999 Cancer Research Campaign 

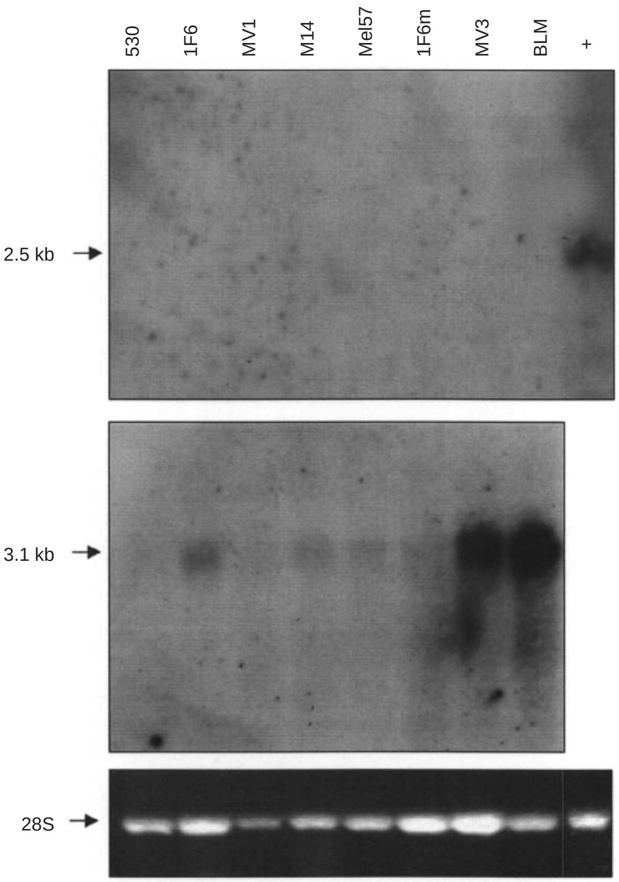

**Figure 2** Northern blot analysis of MMP-1 and MMP-2 expression in human melanoma cell lines. M24met mRNA was used as a positive control for MMP-1. mRNA sizes are indicated (kb)

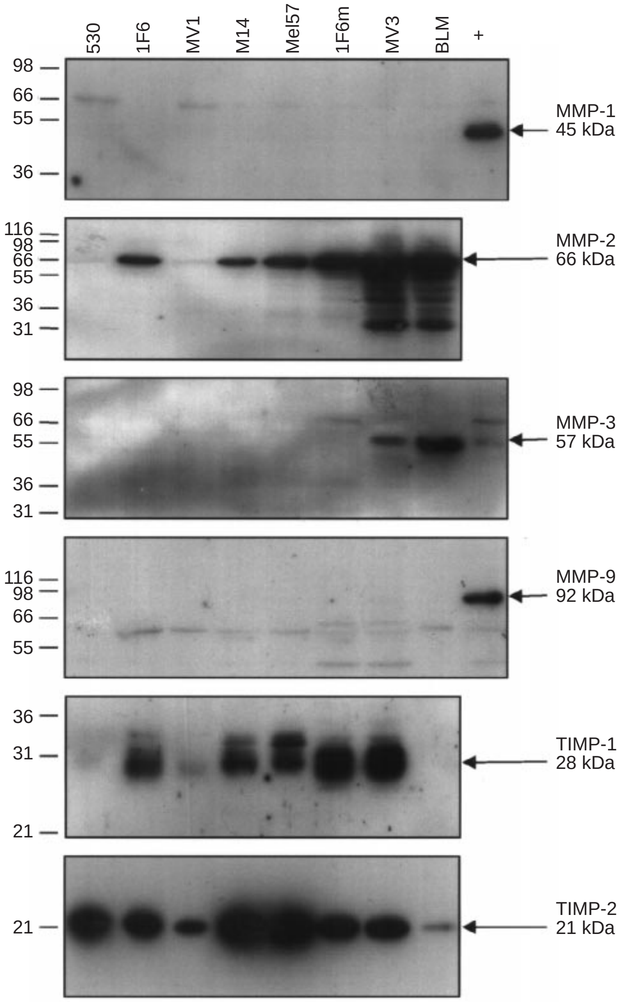

**Figure 3** Western blot analysis of MMP-1, MMP-2, MMP-3, MMP-9, TIMP-1 and TIMP-2 expression in human melanoma cell lines. M24met and HT1080 SFCM were used as positive controls. Position and molecular size of markers are indicated

Addition of p-APMA resulted in an expression of active MMP-2 in all cell lines. Consistent with the Western blot results MMP-9 was not detectable in our melanoma cell line panel (Figure 4).

### MMP and TIMP mRNA in melanoma xenografts

Frozen tissue sections from melanoma xenograft lesions derived from cell lines 530, Mel57, 1F6m, MV3 and BLM were used to determine mRNA expression of MMPs and TIMPs in vivo (Figure 5). The RT-PCR data of 1F6, MV1 and M14 are not shown, because melanin present in the xenografts copurified with the RNA, and caused strong inhibition of the RT-reaction (Westphal et al, manuscript in preparation). Expression of MMP-1 mRNA was found in MV3 and BLM xenografts and, to a lesser extent, in 530 tumours. MMP-2 mRNA was detectable in 1F6m, MV3 and BLM xenografts. MMP-3 and TIMP-1 mRNA were not expressed in any xenografts. A very low MMP-9 mRNA expression was observed in 530 xenografts. TIMP-2 mRNA was detectable in all samples.

### MMP and TIMP protein in melanoma xenografts

We used immunohistochemistry to investigate the expression of MMP and TIMP proteins in vivo. Mice xenografts from cell lines 530, Mel57, 1F6m, MV3 and BLM were stained with polyclonal antibodies. Rat xenografts from 1F6 and BLM were used for immunohistochemistry with mouse monoclonal antibodies. A moderate granular cytoplasmatic staining for MMP-1 was detectable in all tumour cells in 1F6 and BLM xenografts. In these lesions the stromal septa separating tumour cell nests showed also a strong immunoreactivity (data not shown). Tumour cells in 530, 1F6 and Mel57 xenografts were MMP-2-negative, whereas some fibroblasts and tumour surrounding stromal cells were MMP-2-positive (Figure 6A). In 1F6m xenografts a moderate granular and cytoplasmatic staining of tumour cells was found. A heterogeneous strong granular cytoplasmatic staining pattern was observed in tumour cells in MV3 and BLM xenografts (Figure 6B). Remarkably, immunoreactivity of tumour cells increased at the growth or invasive front of 1F6m, MV3 and BLM xenografts (Figure 6C). No staining of tumour cells was observed for MMP-3, MMP-9 and TIMP-1, whereas macrophages of mouse origin surrounding the tumours showed a strong immunoreactivity for both MMP-3 and MMP-9 (Figure 6D). A strong immunoreactivity against TIMP-1 was found in blood vessels of rat tissue adjacent to

 © 1999 Cancer Research Campaign

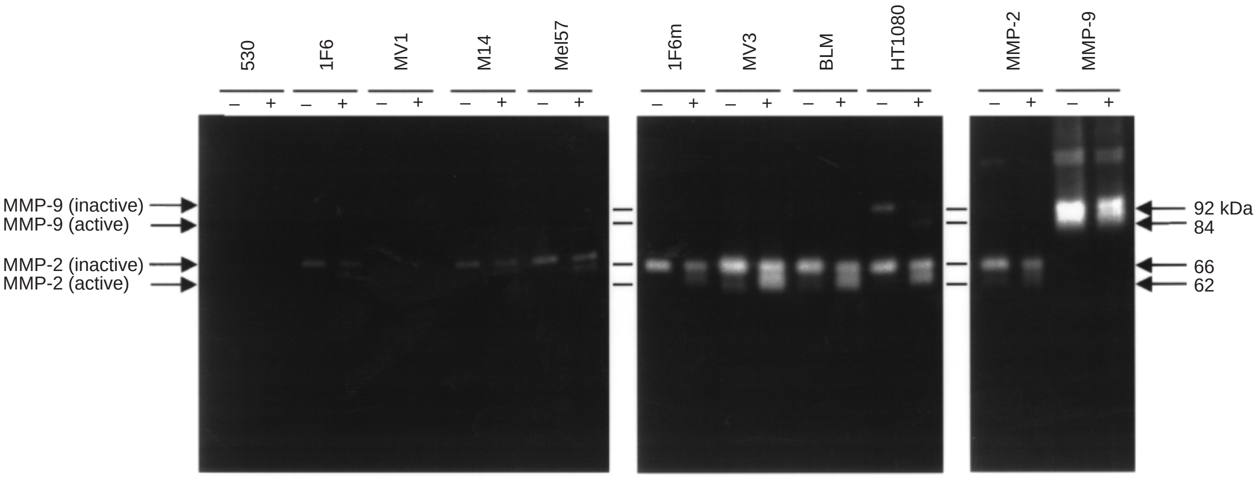

**Figure 4** Zymographic analysis of in vitro MMP-2 and MMP-9 expression. Aliquots of SFCM derived from melanoma cell lines or HT1080 cells (positive control) were incubated in the presence (+) or absence (–) of p-APMA prior to analysis. Purified MMP-2 and MMP-9 protein with (+) and without (–) p-APMA pretreatment were used as controls (right lanes)

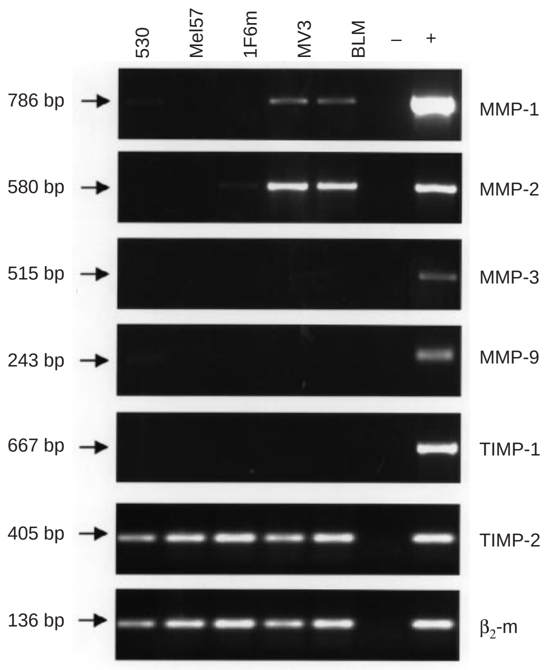

**Figure 5** Expression of MMP-1, MMP-2, MMP-3, MMP-9, TIMP-1 and TIMP-2 mRNA in xenografts. mRNA from M24met or HT1080 cell lines were used as positive controls. PCR was performed at least twice with the same results. Lengths of the PCR products are indicated (bp)

tumours (Figure 6E). In all studied xenografts tumour cells were positive for TIMP-2 protein. The tumour cells in the periphery of necrotic areas stained stronger (Figure 6F).

Finally, we performed zymography to determine the activation status of MMP-2 protein expression in xenografts. The latent form of MMP-2 ($M_r$ 66 000) was detectable in all studied xenografts. Expression of MMP-2 protein in 530 and Mel57 xenografts is due to mouse origin. In these lesions we found a strong immunoreactivity for MMP-2 only in macrophages surrounding the tumours. The $M_r$ 62 000 band representing the activated form of MMP-2, however, showed increased expression levels in MV3 and BLM xenografts (Figure 7).

### DISCUSSION

Cutaneous melanoma is characterized by a high capacity for invasion and metastasis. In this process degradation of the ECM and basement membranes by proteolytic enzymes is an essential step, in which MMPs play an important role (Stetler Stevenson et al, 1993; Mueller, 1996). Overexpression of MMP-1, MMP-2 and MMP-9 has been implicated in the migration and invasion of melanoma cells (MacDougall et al, 1995; Ray and Stetler Stevenson, 1995; Durko et al, 1997). The relative contribution of these enzymes to melanoma invasion and metastasis, however, is not fully understood. We used a well-defined human melanoma xenograft model to investigate the expression profiles of MMP-1, MMP-2, MMP-3, MMP-9, TIMP-1 and TIMP-2, and their correlation to melanoma progression.

Compared with the cultured cell lines where MMP-1 protein was undetectable, expression was up-regulated in xenograft lesions. A moderate staining for MMP-1 was found in xenografts of both the non-metastatic cell line 1F6 and the highly metastatic cell line BLM. A strong immunoreactivity for MMP-1 was detectable in stromal septa surrounding the tumour nests, indicating that host mechanisms may be involved in up-regulated expression of MMP-1 in vivo. It has been demonstrated that expression of MMPs in tumour surrounding stromal cells is an important factor in the process of tumour progression (MacDougall et al, 1995). In another study expression of MMP-1 has been shown to correlate with invasive capacity of melanomas (Woolley et al, 1980). In agreement with this, Durko et al (1997) showed that invasion of human melanoma cells through type VI collagen and a reconstituted basement membrane (Matrigel) was dependent on expression of MMP-1.

An increased expression of MMP-2 has been shown to be associated with tumour progression in several tumour types (Nomura et al, 1995; Lambert et al, 1998). In melanocytic lesions, a correlation between MMP-2 protein expression and decreased architectural organization, increased atypia, and haematogenous

© 1999 Cancer Research Campaign

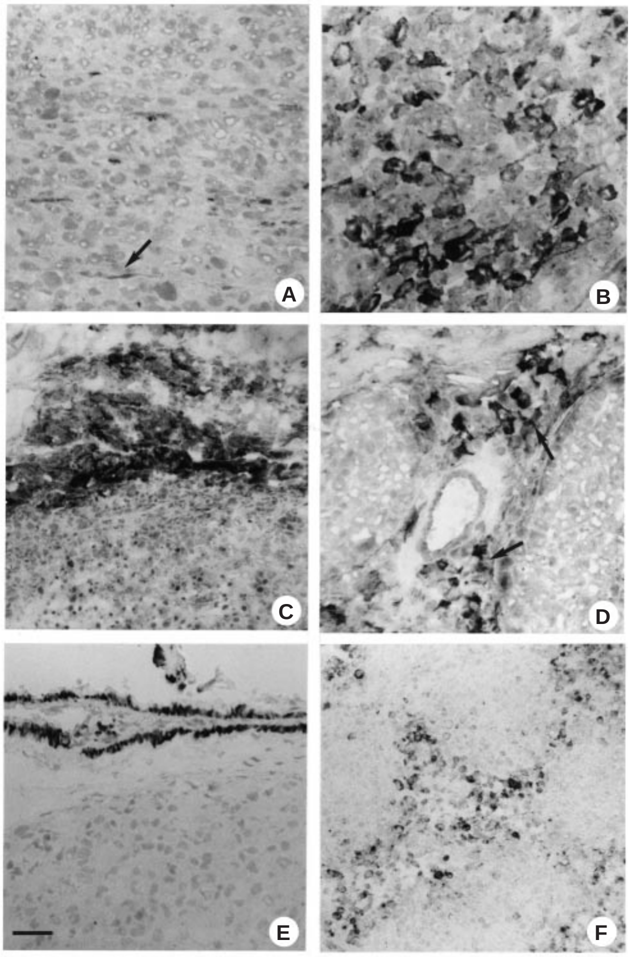

**Figure 6** Immunohistochemical staining of MMPs and TIMPs in xenografts. (**A**) Staining of MMP-2 in 1F6 xenograft. Note MMP-2 expression in fibroblasts (arrow), whereas tumour cells are negative. (**B**) MMP-2 expression in MV3 xenograft. Note heterogeneous strong staining of tumour cells. (**C**) BLM xenograft stained for MMP-2. Note a stronger expression of MMP-2 at the tumour border. (**D**) Staining of MMP-3 in Me 57 xenograft showing a strong positivity of macrophages surrounding the tumour (arrows). (**E**) TIMP-1 expression in BLM xenograft. Note negative staining of tumour cells, whereas smooth muscle cells in the wall of blood vessels stained positive. (**F**) Staining of TIMP-2 in BLM xenograft showing a moderate granular staining of tumour cells. Note that tumour cells in the periphery of necrotic areas stained stronger. Nuclear staining with haematoxylin. Magnification: bar = 30 µm in **A, B, D, E**; bar = 60 µm in **C, F**

metastasis was described (Vaisanen et al, 1996). In another study by the same group (Vaisanen et al, 1998), a positive correlation between high level of MMP-2 protein expression and poor prognosis in melanoma patients was found. Consistent with this, we found clearly elevated expression level of MMP-2 mRNA and protein in the most aggressive cell lines MV3 and BLM as well as in the corresponding xenografts. The discrepancy in the levels of MMP-2 mRNA in vitro, as determined by RT-PCR and Northern blot is most likely due to the fact that the results of RT-PCR represent the end-point of the reaction, making a quantitative comparison of samples impossible. Since the ability of a tumour to activate MMP-2 may be more important than MMP-2 expression per se, we also studied MMP-2 activation status. We found that activated MMP-2 of tumour cells was restricted to the most aggressive cell lines MV3 and BLM, both in vitro and in vivo. In line with this, activation of MMP-2 was shown to correlate with malignant progression in brain tumours (Yamamoto et al, 1996), lung (Tokuraku et al, 1995) and gastric carcinomas (Nomura et al, 1995).

The specific mechanism of proMMP-2 activation is not fully understood. It has been demonstrated that increased expression of MT1-MMP results in activation of MMP-2 on the cell surface, which is required for cell invasion when localized to invadopodia of human melanoma cells (Nakahara et al, 1997). MMP-2 activity, however, is also regulated by the amount of TIMP-2 present (Butler et al, 1998). On one hand, TIMP-2 may act as a specific inhibitor of MMP-2, and has been shown to inhibit tumour growth and tumour cell invasion (Montgomery et al, 1994; Imren et al, 1996; Valente et al, 1998). On the other hand, TIMP-2 may be directly involved in MMP-2 activation by forming a complex with MT1-MMP that functions as a receptor for MMP-2 at the cell surface (Strongin et al, 1995; Butler et al, 1998; Zucker et al, 1998). A coordinated expression of MMP-2, MT1-MMP and TIMP-2 resulting in an activation of MMP-2 was also reported to be correlated with malignant progression in other human cancers (Kanayama et al, 1998; Lambert et al, 1998). We found TIMP-2 mRNA and protein expressed in all cell lines in vitro and in vivo. Whether in our xenograft model TIMP-2 acts as a MMP-2 inhibitor, as a MMP-2 co-activator, or both, remains to be determined.

The role of MMP-9 in melanoma progression is unclear. Whereas we found virtually no expression of MMP-9 mRNA and protein in tumour cells in vitro and in vivo, MMP-9 expression

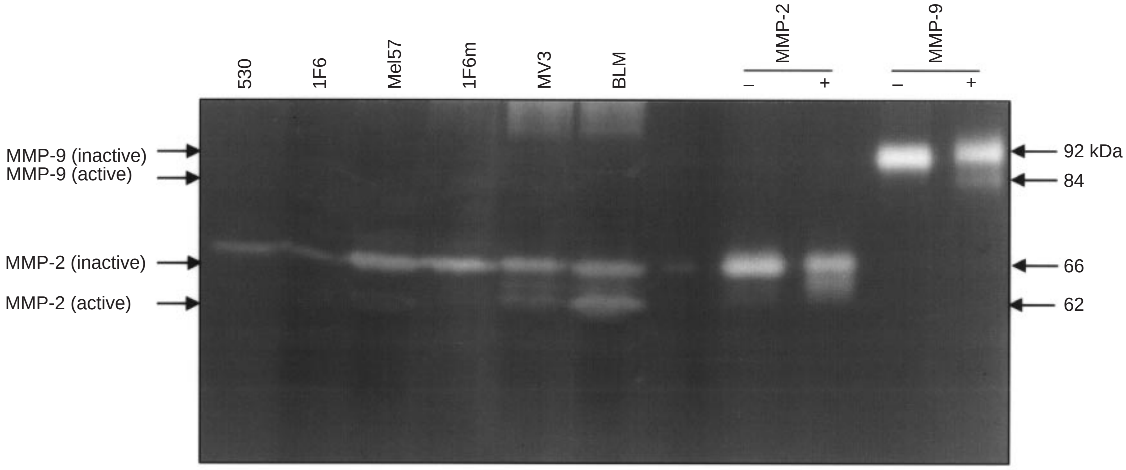

**Figure 7** Zymographic analysis of xenograft tissue extracts. MV3 and BLM xenografts express increased levels of active MMP-2

 *© 1999 Cancer Research Campaign*

was described in seven melanoma cell lines derived from advanced stage melanoma, but not from early stage melanoma (MacDougall et al, 1995). In contrast, van den Oord et al (1997) found MMP-9 expression in human melanoma cells in the radial growth phase of primary melanoma with thickness < 1.6 mm, but not in melanoma metastases. In line with this, the lack of MMP-9 expression in our panel of cell lines may be attributed to the fact that all lines are derived from human melanoma metastases. Our data on MMP-9 expression taken together with several reports based on immunohistochemistry and in situ hybridization indicate that MMP-9 may not expressed by tumour cells but predominantly by tumour surrounding stromal cells (Heppner et al, 1996; Nielsen et al, 1996; Sugiura et al, 1998).

To the best of our knowledge the expression profile of MMP-3 in melanoma has not yet been studied. MMP-3 protein was shown to be expressed by the highly metastatic cell lines MV3 and BLM, suggesting a possible role for MMP-3 in tumour cell invasion and metastasis. In agreement with this, Sreenath et al (1992) showed that high levels of MMP-3 expression were associated with metastatic potential of transformed rat embryo cells. In xenograft lesions we found no expression of MMP-3 protein in tumour cells, whereas a strong immunoreactivity was observed in macrophages and tumour surrounding stromal cells. A similar stromal cell expression pattern has also been described in other tumour types as colorectal cancer (Newell et al, 1994). Our observations on MMP-3 and MMP-9 expression by macrophages and fibroblasts supports the possible involvement of these cells in melanoma progression. Studies on MMP expression by stromal cells in human melanoma are currently in progress.

TIMP-1 serves as a regulatory element of MMP-9 activation (Gomez et al, 1997; Nagase, 1997), and increased expression of TIMP-1 inhibits tumour growth, invasion and metastasis of melanoma cells in vitro and in vivo (Khokha et al, 1992; Khokha, 1994). In our study we found that TIMP-1 was expressed by all cell lines, but no expression was detectable in xenografts, indicating that TIMP-1 expression is down-regulated in vivo. Therefore, the role of this molecule in tumour progression in our xenograft model appears to be limited.

Although the specific role of the different MMPs in tumour progression, and the specific activation mechanism of the MMPs in vivo still remains unclear, our study shows that an increased expression of activated MMP-2 correlates with metastatic behaviour in our xenograft model. Our data taken together with previous studies strongly implicate an important role for activated MMP-2 in melanoma progression. Studies on specifically blocking MMP-2 activity may lead to novel strategies in the systemic therapy of malignant melanoma.

### ACKNOWLEDGEMENTS

We thank R Hanemaaijer (Department of Vascular and Connective Tissue Research, Gaubius Laboratory TNO-PG, Leiden, The Netherlands) for providing DNA-probes, and W van Geloof for expert technical assistence. This work was supported by the Deutsche Forschungsgemeinschaft Grant Ho 2004/1–1

### REFERENCES

Ahonen M, Baker AH and Kahari VM (1998) Adenovirus-mediated gene delivery of tissue inhibitor of metalloproteinases-3 inhibits invasion and induces apoptosis in melanoma cells. *Cancer Res* **58**: 2310–2315

Butler GS, Butler MJ, Atkinson SJ, Will H, Tamura T, van-Westrum SS, Crabbe T, Clements J, d'Ortho MP and Murphy G (1998) The TIMP2 membrane type 1 metalloproteinase 'receptor' regulates the concentration and efficient activation of progelatinase A. A kinetic study. *J Biol Chem* **273**: 871–880

Chambers AF and Matrisian LM (1997) Changing views of the role of matrix metalloproteinases in metastasis. *J Natl Cancer Inst* **89**: 1260–1270

Coussens LM and Werb Z (1996) Matrix metalloproteinases and the development of cancer. *Chem Biol* **3**: 895–904

Durko M, Navab R, Shibata HR and Brodt P (1997) Suppression of basement membrane type IV collagen degradation and cell invasion in human melanoma cells expressing an antisense RNA for MMP-1. *Biochim Biophys Acta* **1356**: 271–280

Ferrier CM, van Geloof WL, de Witte HH, Kramer MD, Ruiter DJ and van Muijen GN (1998) Epitopes of components of the plasminogen activation system are re-exposed in formalin-fixed paraffin sections by different retrieval techniques. *J Histochem Cytochem* **46**: 469–476

Gomez DE, Alonso DF, Yoshiji H and Thorgeirsson UP (1997) Tissue inhibitors of metalloproteinases: structure, regulation and biological functions. *Eur J Cell Biol* **74**: 111–122

Grant GM, Cobb JK, Castillo B and Klebe RJ (1996) Regulation of matrix metalloproteinases following cellular transformation. *J Cell Physiol* **167**: 177–183

Heppner KJ, Matrisian LM, Jensen RA and Rodgers WH (1996) Expression of most matrix metalloproteinase family members in breast cancer represents a tumor-induced host response. *Am J Pathol* **149**: 273–282

Imren S, Kohn DB, Shimada H, Blavier L and DeClerck YA (1996) Overexpression of tissue inhibitor of metalloproteinases-2 retroviral-mediated gene transfer in vivo inhibits tumor growth and invasion. *Cancer Res* **56**: 2891–2895

Kanayama H, Yokota K, Kurokawa Y, Murakami Y, Nishitani M and Kagawa S (1998) Prognostic values of matrix metalloproteinase-2 and tissue inhibitor of metalloproteinase-2 expression in bladder cancer. *Cancer* **82**: 1359–1366

Khokha R (1994) Suppression of the tumorigenic and metastatic abilities of murine B16-F10 melanoma cells in vivo by the overexpression of the tissue inhibitor of the metalloproteinases-1. *J Natl Cancer Inst* **86**: 299–304

Khokha R, Zimmer MJ, Wilson SM and Chambers AF (1992) Up-regulation of TIMP-1 expression in B16-F10 melanoma cells suppresses their metastatic ability in chick embryo. *Clin Exp Metastasis* **10**: 365–370

Lambert K, Machein U, Machein MR, Conca W, Peter HH and Volk B (1998) Expression of matrix metalloproteinases and their tissue inhibitors in human brain tumors. *Am J Pathol* **153**: 429–437

MacDougall JR and Matrisian LM (1995) Contribution of tumor and stromal matrix metalloproteinases to tumor progression, invasion and metastasis. *Cancer Metastasis Rev* **14**: 351–362

MacDougall JR, Bani MR, Lin Y, Rak J and Kerbel RS (1995) The 92-kDa gelatinase B is expressed by advanced stage melanoma cells: suppression by somatic cell hybridization with early stage melanoma cells. *Cancer Res* **55**: 4174–4181

Montgomery AM, Mueller BM, Reisfeld RA, Taylor SM and DeClerck YA (1994) Effect of tissue inhibitor of the matrix metalloproteinases-2 expression on the growth and spontaneous metastasis of a human melanoma cell line. *Cancer Res* **54**: 5467–5473

Mueller BM (1996) Different roles for plasminogen activators and metalloproteinases in melanoma metastasis. *Curr Top Microbiol Immunol* **213**: 65–80

Nagase H (1997) Activation mechanisms of matrix metalloproteinases. *Biol Chem* **378**: 151–160

Nakahara H, Howard L, Thompson EW, Sato H, Seiki M, Yeh Y and Chen WT (1997) Transmembrane/cytoplasmic domain-mediated membrane type 1-matrix metalloprotease docking to invadopodia is required for cell invasion. *Proc Natl Acad Sci USA* **94**: 7959–7964

Newell KJ, Witty JP, Rodgers WH and Matrisian LM (1994) Expression and localization of matrix-degrading metalloproteinases during colorectal tumorigenesis. *Mol Carcinog* **10**: 199–206

Nielsen BS, Timshel S, Kjeldsen L, Sehested M, Pyke C, Borregaard N and Dano K (1996) 92 kDa type IV collagenase (MMP-9) is expressed in neutrophils and macrophages but not in malignant epithelial cells in human colon cancer. *Int J Cancer* **65**: 57–62

Nomura H, Sato H, Seiki M, Mai M and Okada Y (1995) Expression of membrane-type matrix metalloproteinase in human gastric carcinomas. *Cancer Res* **55**: 3263–3266

Ray JM and Stetler Stevenson WG (1995) Gelatinase A activity directly modulates melanoma cell adhesion and spreading. *EMBO J* **14**: 908–917

© 1999 Cancer Research Campaign

Sato H, Takino T, Okada Y, Cao J, Shinagawa A, Yamamoto E and Seiki M (1994) A matrix metalloproteinase expressed on the surface of invasive tumour cells. *Nature* **370**: 61–65

Sreenath T, Matrisian LM, Stetler SW, Gattoni CS and Pozzatti RO (1992) Expression of matrix metalloproteinase genes in transformed rat cell lines of high and low metastatic potential. *Cancer Res* **52**: 4942–4947

Stetler Stevenson WG, Aznavoorian S and Liotta LA (1993) Tumor cell interactions with the extracellular matrix during invasion and metastasis. *Annu Rev Cell Biol* **9**: 541–573

Strongin AY, Collier I, Bannikov G, Marmer BL, Grant GA and Goldberg GI (1995) Mechanism of cell surface activation of 72-kDa type IV collagenase. Isolation of the activated form of the membrane metalloproteinase. *J Biol Chem* **270**: 5331–5338

Sugiura Y, Shimada H, Seeger RC, Laug WE and DeClerck YA (1998) Matrix metalloproteinases-2 and -9 are expressed in human neuroblastoma: contribution of stromal cells to their production and correlation with metastasis. *Cancer Res* **58**: 2209–2216

Tokuraku M, Sato H, Murakami S, Okada Y, Watanabe Y and Seiki M (1995) Activation of the precursor of gelatinase A/72 kDa type IV collagenase/MMP-2 in lung carcinomas correlates with the expression of membrane-type matrix metalloproteinase (MT-MMP) and with lymph node metastasis. *Int J Cancer* **64**: 355–359

Vaisanen A, Tuominen H, Kallioinen M and Turpeenniemi Hujanen T (1996) Matrix metalloproteinase-2 (72 kD type IV collagenase) expression occurs in the early stage of human melanocytic tumour progression and may have prognostic value. *J Pathol* **180**: 283–289

Vaisanen A, Kallioinen M, Taskinen PJ and Turpeenniemi-Hujanen T (1998) Prognostic value of MMP-2 immunoreactive protein (72 kD type IV collagenase) in primary skin melanoma. *J Pathol* **186**: 51–58

Valente P, Fassina G, Melchiori A, Masiello L, Cilli M, Vacca A, Onisto M, Santi L, Stetler Stevenson WG and Albini A (1998) TIMP-2 over-expression reduces invasion and angiogenesis and protects B16F10 melanoma cells from apoptosis. *Int J Cancer* **75**: 246–253

van den Oord JJ, Paemen L, Opdenakker G and de Wolf Peeters C (1997) Expression of gelatinase B and the extracellular matrix metalloproteinase inducer EMMPRIN in benign and malignant pigment cell lesions of the skin. *Am J Pathol* **151**: 665–670

van Muijen GN, Cornelissen LM, Jansen CF, Figdor CG, Johnson JP, Brocker EB and Ruiter DJ (1991*a*) Antigen expression of metastasizing and non-metastasizing human melanoma cells xenografted into nude mice. *Clin Exp Metastasis* **9**: 259–272

van Muijen GN, Jansen KF, Cornelissen IM, Smeets DF, Beck JL and Ruiter DJ (1991*b*) Establishment and characterization of a human melanoma cell line (MV3) which is highly metastatic in nude mice. *Int J Cancer* **48**: 85–91

Westphal JR, van't Hullenaar RG, van der Laak JA, Cornelissen IM, Schalkwijk LJ, van Muijen GN, Wesseling P, de Wilde PC, Ruiter DJ and de Waal RM (1997) Vascular density in melanoma xenografts correlates with vascular permeability factor expression but not with metastatic potential. *Br J Cancer* **76**: 561–570

Wooley DE and Grafton CA (1980) Collagenase immunolocalization studies of cutaneous secondary melanomas. *Br J Cancer* **42**: 260–265

Yamamoto M, Mohanam S, Sawaya R, Fuller GN, Seiki M, Sato H, Gokaslan ZL, Liotta LA, Nicolson GL and Rao JS (1996) Differential expression of membrane-type matrix metalloproteinase and its correlation with gelatinase A activation in human malignant brain tumors in vivo and in vitro. *Cancer Res* **56**: 384–392

Zucker S, Drews M, Conner C, Foda HD, DeClerck YA, Langley KE, Bahou WF, Docherty AJP and Cao J (1998) Tissue inhibitor of metalloproteinase-2 (TIMP-2) binds to the catalytic domain of the cell surface receptor, membrane type 1-matrix metalloproteinase 1 (MT1-MMP). *J Biol Chem* **273**: 1216–1222

*© 1999 Cancer Research Campaign*
